# The KT Jeang Retrovirology prize 2017: Michael Emerman

**DOI:** 10.1186/s12977-017-0362-5

**Published:** 2017-06-21

**Authors:** Retrovirology Editorial

**Affiliations:** London, UK


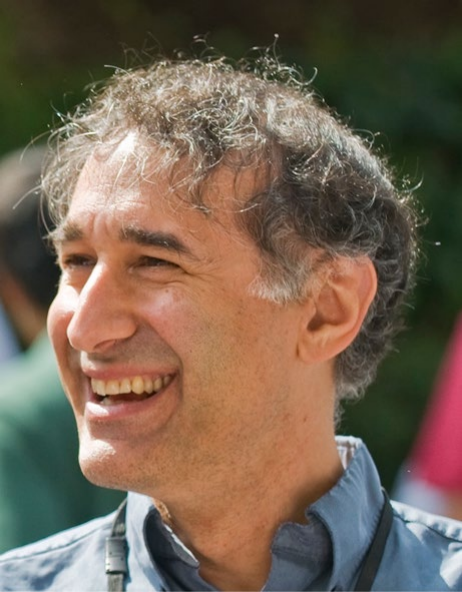


Michael Emerman

Michael Emerman graduated from the Ohio State University in 1981 with an undergraduate degree in Biochemistry. He began his career in retrovirology when he entered the lab of Howard Temin at the University of Wisconsin-Madison for his Ph.D. studies. Howard Temin won the Nobel Prize in 1975 for his provirus hypothesis and the discovery of reverse transcriptase, along with David Baltimore and Renato Dulbecco [1].

At the time Emerman joined the Temin lab in 1982, the development of retroviruses as vectors for transducing foreign genes into cells was new technology. The lab had made retroviral vectors based on the genome of spleen necrosis virus (SNV), a retrovirus of ducks that decades later was shown to be a likely contaminant of malaria challenge experiments [2]. A previous postdoc in the lab had inserted the mouse alpha-globin gene into one of these vectors to show they could be used to generate fully spliced cDNA from a cellular gene with introns [3]. Emerman was given the project of showing whether or not alpha-globin protein was made. After some initial excitement based on finding “red cells” that ended up being bits of rubber from the disintegrating red pipet bulb, he found, instead, that nearly all of the integrated proviruses contained deletions involving the internal promoter as well as point mutations [4]. In subsequent studies, he designed retroviral vectors to more directly test the effects of integration on promoter activity, and found that often either the promoter in the long terminal repeat (LTR) or the internal promoter was silenced in an epigenetic and reversible fashion [5, 6]. This finding has current implications for the epigenetic control of latent proviruses in HIV Cure research. Another of the major outcomes of his thesis work was the realization that one could use single cycle retrovirus vectors to precisely measure the rates of mutations, recombination, and other genetic events in retroviruses—used with increasing levels of sophistication by following generations of Temin labmates, e.g. [7–9].

During the time of his thesis work, 1981–1986, the first AIDS cases were described in California and New York, and the retrovirus now known as HIV-1 was isolated. Emerman decided to do his postdoc in the lab of Luc Montagnier, who was awarded the Nobel Prize in 2005 along with Francois Barrie-Sinoussi and Harald zur Hausen. When he arrived at the Pasteur Institute in Paris in the summer of 1986, HIV-2 had recently been identified [10], and the group was making full-length molecular clones of HIV-2 and he participated in work to compare their sequence and biology to HIV-1 [11, 12]. In other work from that time, he and his colleagues mapped a site in gp120 necessary for binding to CD4 [13], showed that the Vpx protein had a phenotype in primary cells [14], and showed that the *rev* gene was involved in nuclear export of viral RNA [15]. This last work was done about the same time that Mike Malim in Bryan Cullen’s lab made a similar observation [16].

Emerman started his own lab at the Fred Hutchinson Cancer Research Center in Seattle, Washington in 1989 where they had recently been awarded a program project grant to build a BL2/3 lab to work with infectious HIV. In those days, assays determining viral titers for HIV or counting infected cells were neither quick nor easy. One of the first things his lab did was to create an indicator cell line called the MAGI cells (multinuclear activation of (beta)-galactosidase cells) that allowed one to count infected cells. They also used this assay to obtain the first measures of the particle to infectivity ratio of HIV-1 [17]. While this was not the first indicator cell line made, it was the most widely used for many years because it was distributed without restrictions through NIH AIDS Reagent Program to over 500 labs in over 30 countries, although now mostly supplanted by newer and better versions based in similar technology [18].

One paradox of retrovirus research was the ability of HIV (and other lentiviruses such as Visna virus) to infect terminally differentiated macrophages [19, 20] because it contrasted with much earlier work from Howard Temin showing that other retroviruses required cell division [21]. Emerman’s lab used their MAGI cell assay to demonstrate that ability of HIV to infect non-cycling cells was not limited to macrophages [22], and that the difference between lentiviruses and most other retroviruses is due to the need for other retroviruses to pass through mitosis while HIV infection is independent of mitosis [23, 24]. Such findings have been of utility in the development of lentiviral vectors for gene transfer into non-dividing or slowly dividing cells such as stem cells.

Emerman’s lab, as well as several many others, put forth evidence for different viral factors that could mediate the ability of HIV to infect non-cycling cells. These included MA, IN, Vpr, and the cPPT, although eventually, none of theses factors could be reproduced when more sensitive and quantitative assays came around, e.g. [25, 26]. However, Masahiro Yamashita, a postdoctoral fellow in the Emerman lab, used chimeric HIV/MLV viruses to show that the CA protein of HIV was the dominant viral factor necessary for entry of HIV into the nucleus in the absence of mitosis, and later followed this up by identifying particular CA mutations that affected this phenotype [27–29]. These findings led to others to exciting ongoing work focusing on the role of CA in HIV nuclear import and its targeting by host cell factors, e.g. [29–34].

During the course of mapping phenotypes of HIV that could be attributed to its accessory proteins, Emerman had started some long term cultures of T cells infected with HIV either wt virus or virus mutated in its *vpr* gene. Nearly all of the cells in wells infected with wt virus died, while those in the wells with a *vpr* mutant went on to establish chronic infections. Eventually, a few cells grew out of the wt infected cultures (after he had forgotten about them in the incubator for a few weeks), and, by sequence analysis, he found that all of them contained a mutation in *vpr*. This led to the realization that Vpr caused an arrest in the cell cycle in the G2 phase [35]. Similar work was published soon after by other labs [36–38]. While Vpr appears to modestly increase LTR activity due to its cell cycle effects [39, 40] and its ability to cause a DNA damage response is conserved among lentiviral homologs [41], the role of Vpr in the HIV-1 lifecycle is still an area under exploration.

The Fred Hutchinson Cancer Research Center has a tradition of very interactive faculty talks where preliminary work and ideas are discussed. It was at one of these retreats that Emerman talked about the relatively recent discovery by the Malim group of the host factor antagonized by HIV-1 Vif, APOBEC3G [42]. Harmit Malik was a new faculty hire whose research focus was on “genetic conflict”, or the process by which different genetic elements try to gain an advantage over each other. Malik suggested that the Vif-APOBEC3G interaction would be a great system to look for signs of this genetic “arms race” between viruses and host proteins. Malik and Emerman enlisted Malik’s first postdoc, Sara Sawyer, in this project to look for evidence of positive selection (a signature of genetic conflict) in APOBEC3 proteins. The results were very dramatic—there was positive selection in APOBEC3G throughout primate evolution including very ancient signs of this arms race [43]. This work led Emerman and Malik to the development of the concept of “paleovirology” as a means to discover the effects of ancient pathogenic challenges on modern innate immunity [44].

Despite finding much evidence of a genetic arms race in APOBEC3G, the first study did not find any evidence to support the initial assumption that Vif had driven this evolution [43]. It was later discovered this was due to the fact that the initial study did not look in the right species of monkeys when a graduate student in the Emerman lab found evidence for ongoing genetic conflicts between APOBEC3G and Vif in the African Monkeys who are infected with SIVagm [45]. The Emerman lab subsequently found additional instances of positive selection in APOEC3G that were escape mutations from antagonism from Vif proteins [46]. This approach allowed the Emerman and his colleagues to conclude that primate lentiviruses are much older than the oldest date that had been previously calculated [47] being at least 5–10 million years old [46, 48]. A parallel study by Welkin Johnson’s group came to a similar age using the genetic conflict between SIV CA and the host restriction factor Trim5 [49].

Since their initial study on APOBEC3G, Harmit Malik and Michael Emerman have collaborated on over 20 other papers together where they have used their evolutionary approach to study host antiviral genes. They have used positive selection to identify sites of interaction between restriction factors and viral proteins, identify novel activities of known restriction factors, and find additional evidence for ancient pathogens that drove selection on restriction factors, in particular using Trim5 and other Trim proteins [50–54], Tetherin [55], Zinc Antiviral Protein [56], Mx proteins [57, 58], APOBEC3 proteins [59–61], and SAMHD1 [62]. They have also used human polymorphism data and comparison between primates to understand “holes” in the human innate immune system where some, most, or sometimes all, humans are ill-adapted to cope with lentiviral infections. These include functionally important human polymorphisms in Trim5 [63], APOBEC3D [59, 64], APOBEC3C [64, 65], and APOBEC3H [60, 66–68]. Finally, the Emerman lab has used this approach to understand forces that underlie the ancient evolutionary history of lentiviral lineages leading to HIV-1 including the adaptation of SIV to hominid APOBEC3 proteins that involved the loss of SAMHD1 antagonism [69], the gain of SAMHD1 antagonism by Vpr and Vpx proteins [62] (as well as changes in specificity of SAMHD1 antagonism in lentiviral evolution [70, 71]) and the adaptation of HIV-1 to human Tetherin [55]. Additional work has focused on restriction factors that protect hominids from other lentiviral infections [72].

Emerman has been an editor for Virology since 2002 and the Editor-in-Chief since 2013. He is also an Associate Editor for PLOS Pathogens. He teaches a graduate course in Virology every other year called “Human Pathogenic Viruses” which focuses on replication, evolution/ecology, and pathogenesis of major human virus families. He was awarded the Ohio State University Center for Retrovirus Research Distinguished Career Award, an NIH Merit Award, and Elected Fellow in the American Academy of Microbiology. He has trained over 30 graduate students and postdocs in retrovirology, and the prize of which he is most proud is the James McDougall Mentoring Award nominated by his former trainees.
